# Prediction of major postoperative events after non-cardiac surgery for people with kidney failure: derivation and internal validation of risk models

**DOI:** 10.1186/s12882-023-03093-6

**Published:** 2023-03-10

**Authors:** Tyrone G. Harrison, Brenda R. Hemmelgarn, Matthew T. James, Simon Sawhney, Braden J. Manns, Marcello Tonelli, Shannon M Ruzycki, Kelly B. Zarnke, Todd A. Wilson, Deirdre McCaughey, Paul E. Ronksley

**Affiliations:** 1 Department of Medicine, University of Calgary, Calgary, AB Canada; 2 O’Brien Institute for Public Health, Cumming School of Medicine, University of Calgary, Calgary, AB Canada; 3 Department of Medicine, University of Alberta, Edmonton, AB Canada; 4 Department of Community Health Sciences, Cumming School of Medicine, University of Calgary, Cal Wenzel Precision Health Building, Room 3E18B, 3280 Hospital Drive NW, Calgary, AB T2N 4Z6 Canada; 5 Cumming School of Medicine, Libin Cardiovascular Institute, University of Calgary, Calgary, AB Canada; 6 Aberdeen Centre for Health Data Sciences, University of Aberdeen, Aberdeen, Scotland UK; 7 National Health Service, Grampian, Aberdeen, Scotland UK

**Keywords:** Kidney disease, Perioperative, Surgery, Risk prediction, Outcomes

## Abstract

**Background:**

People with kidney failure often require surgery and experience worse postoperative outcomes compared to the general population, but existing risk prediction tools have excluded those with kidney failure during development or exhibit poor performance. Our objective was to derive, internally validate, and estimate the clinical utility of risk prediction models for people with kidney failure undergoing non-cardiac surgery.

**Design, setting, participants, and measures:**

This study involved derivation and internal validation of prognostic risk prediction models using a retrospective, population-based cohort. We identified adults from Alberta, Canada with pre-existing kidney failure (estimated glomerular filtration rate [eGFR] < 15 mL/min/1.73m^2^ or receipt of maintenance dialysis) undergoing non-cardiac surgery between 2005–2019. Three nested prognostic risk prediction models were assembled using clinical and logistical rationale. Model 1 included age, sex, dialysis modality, surgery type and setting. Model 2 added comorbidities, and Model 3 added preoperative hemoglobin and albumin. Death or major cardiac events (acute myocardial infarction or nonfatal ventricular arrhythmia) within 30 days after surgery were modelled using logistic regression models.

**Results:**

The development cohort included 38,541 surgeries, with 1,204 outcomes (after 3.1% of surgeries); 61% were performed in males, the median age was 64 years (interquartile range [IQR]: 53, 73), and 61% were receiving hemodialysis at the time of surgery. All three internally validated models performed well, with c-statistics ranging from 0.783 (95% Confidence Interval [CI]: 0.770, 0.797) for Model 1 to 0.818 (95%CI: 0.803, 0.826) for Model 3. Calibration slopes and intercepts were excellent for all models, though Models 2 and 3 demonstrated improvement in net reclassification. Decision curve analysis estimated that use of any model to guide perioperative interventions such as cardiac monitoring would result in potential net benefit over default strategies.

**Conclusions:**

We developed and internally validated three novel models to predict major clinical events for people with kidney failure having surgery. Models including comorbidities and laboratory variables showed improved accuracy of risk stratification and provided the greatest potential net benefit for guiding perioperative decisions. Once externally validated, these models may inform perioperative shared decision making and risk-guided strategies for this population.

**Supplementary Information:**

The online version contains supplementary material available at 10.1186/s12882-023-03093-6.

## Introduction

Kidney failure is marked by an estimated glomerular filtration rate (eGFR) less than 15 mL/min/1.73m^2^ or receipt of chronic kidney replacement therapy [1], and is associated with high health care utilization and poor health outcomes [2–5]. Surgery is an important component of the health care burden for people with kidney failure, occurring up to 16 times more often than among people with normal kidney function [6]. People with kidney failure additionally have higher risks of death, cardiovascular events, and other complications following inpatient and ambulatory surgery [7–9].

Characterization of the perioperative risk through risk prediction models may inform several decisions in the perioperative period depending on the clinical context, surgical indications and technique, and goals of the procedure (ranging from life-preserving to cosmetic purposes) [10]. Further, risk prediction models may also be useful for guiding allocation of scarce surgical resources, planning additional perioperative interventions for the highest risk individuals who may warrant more intensive care strategies, and for risk adjustment when benchmarking outcomes of surgical centers or individuals. As an example, postoperative cardiac monitoring strategies have been suggested by major perioperative guidelines based on estimated risk from the Revised Cardiac Risk Index (RCRI), which predicts risk of postoperative events within 30 days of surgery [11]. Tools such as the RCRI are widely used, but their value is limited in estimating the risk of major postoperative events for people with kidney failure, because they excluded people with kidney failure during development, exhibit poor performance when applied to people with kidney failure, and omit kidney failure specific variables that are associated with risk [12].

In this study, we derived and internally validated a series of multivariable risk prediction models to predict the risk of major cardiac events or death within 30 days of non-cardiac surgery for people with kidney failure. We then estimated the clinical utility of these models using decision curve analysis.

## Methods

### Study design and source of data

We used the Alberta Kidney Disease Network (AKDN) database to derive a retrospective, population-based cohort using linked administrative health, laboratory, and kidney failure datasets from Alberta, Canada [13]. These data were used to derive and internally validate our multivariable risk prediction models. We conducted this study using a prespecified protocol in accordance with the transparent reporting of a multivariable prediction model for individual prognosis or diagnosis (TRIPOD) Checklist for Prediction model development (Supplementary Table 1). The University of Calgary and the University of Alberta granted ethics approval and waived the need for informed consent.

### Participants

We included all adults (≥ 18 years) with an inpatient or ambulatory surgery performed between April 1 2005 and February 28 2019 in Alberta, Canada. Surgeries were identified using the Canadian Classification of Health Interventions (CCI) coding [14], which is a standardized coding system for procedures. Radiologic or non-surgical procedures were excluded (e.g., endoscopy, hemodialysis catheter insertion, arteriovenous [AV] fistulogram, etc.). Further, we included only those with preoperative kidney failure, defined as an eGFR < 15 mL/min/1.73m^2^ or receiving hemodialysis or peritoneal dialysis for at least 90 days as an outpatient before the index surgical procedure. For non-dialysis participants, at least two outpatient measures of serum creatinine between 7–365 days were necessary prior to surgery to avoid misclassification of people with preoperative acute kidney injury, per a validated algorithm [15]. We estimated eGFR using the Chronic Kidney Disease Epidemiology Collaboration (CKD-EPI) equation without including the Black race coefficient [16]. We excluded people that left Alberta within 30 days of their surgery, and those without available demographic data.

### Outcome

The outcome was a composite of all-cause mortality or major cardiac events within 30 days of the index surgical procedure, which included acute myocardial infarction (AMI) and non-fatal ventricular arrhythmias identified using validated algorithms (Supplementary Table 2). This composite outcome is similar to other postoperative risk tools and was informed by perioperative cardiac risk assessment guidelines [11].

### Predictors

We identified candidate predictors from the literature and input from clinicians with expertise in kidney failure and perioperative medicine. The final list of variables included demographics of age and sex. Surgeries were categorized into 11 surgery types based on CCI codes, including categories that are specific to people with kidney failure (kidney transplant, peritoneal dialysis catheter insertion, and AV fistula creation). Surgery setting was classified using the administrative data as ambulatory elective, inpatient elective, or inpatient urgent/emergent. We considered comorbidities of previous AMI, cancer, chronic pulmonary disease, dementia, diabetes, heart failure, hypertension, liver disease, obesity, peripheral vascular disease, and stroke. These were defined using validated algorithms of International Statistical Classification of Diseases and Related Health Problems Ninth and Tenth Revision (ICD-9-CM and ICD-10-CA) codes [17] with an unrestricted lookback period for permanent conditions and 3 years for temporary conditions (Supplementary Tables 3 and 4). Kidney failure treatment modality was categorized as non-dialysis, hemodialysis, or peritoneal dialysis. Preoperative outpatient serum albumin (in g/L) and serum hemoglobin (in g/L) within the year before surgery were included as candidates. There were no missing values for variables except for albumin (15%) and hemoglobin (0.2%), which were imputed using multivariable normal regression with an iterative Markov chain Monte Carlo method.

### Sample Size

We performed sample size calculations recommended for binary outcomes [18], using the ‘pmsampsize’ module in Stata software version 16 and 17 (StataCorp) [19]. For these calculations, we used an expected R^2^ of 0.072 from a recent study with a similar model [20], expected outcome incidences ranging from 1.7% for ambulatory surgery [9] and 8.0% for inpatient surgery [20], and the maximum candidate predictor parameters of 32. The minimum sample size ranged from 3838 to 3881 participants with 66 to 308 events depending on the incidence values used for the expected outcomes (Supplementary Table 5).

### Statistical analysis methods

For all analyses we used Stata software v16 and 17 (StataCorp). Baseline characteristics were summarized with counts for categorical or dichotomous variables, and medians and interquartile ranges (IQR) for continuous variables. We examined all candidate variables for collinearity and did not identify any. Age was centred at 18 years, and then examined for a non-linear relationship with our outcome, first visually and with the ‘lincheck’ and ‘boxtid’ modules in Stata [21, 22]. Serum hemoglobin and albumin were examined prior to imputation, and there was no evidence of non-linearity for any of the continuous variables.

We developed three nested logistic regression models with prespecified sets of variables with increasing complexity, taking into consideration their availability in different perioperative settings. Standard errors of each variable included in the models were adjusted for clustering of surgeries within cohort participants. We first developed a minimum model with only age, sex, surgery type, surgery setting, and kidney failure type. We then developed a second model including all variables from the first model with the addition of comorbidities, which would likely only be available in perioperative consultative settings. Our final model included all variables included in the second model and also preoperative albumin and hemoglobin, as these may be available in some but not all perioperative settings. For each model, we estimated the apparent discrimination with c-statistics (with accompanying 95% confidence intervals [CI]), and the area under the precision recall curve (AUPRC), as it is more informative with infrequent outcomes [23]. We internally validated each model using bootstrap resampling with 1000 samples, using the ‘bsvalidation’ package in Stata [24]. We compared bootstrap model discrimination using c-statistics, and calibration with calibration slopes and intercepts, and with visual inspection of calibration curves across deciles of predicted risk. An ideal calibration slope is 1, and intercept is 0 [25]. To examine whether performance estimates were robust to the potential bias introduced by including multiple procedures per participant, we examined the performance of all model predictions limited to one surgery per participant.

We compared model fit with Akaike’s and Bayesian Information Criteria (AIC and BIC). To evaluate whether there was an incremental improvement in reclassification of risk in more complex models, we estimated the categorical Net Reclassification Improvement (NRI) and Integrated Discrimination Improvement (IDI) index. If well-calibrated, estimating the NRI for a model with added variables to a simpler nested model may provide insight into whether the more complex model improved overall prediction [26]. We estimated the NRI by examining reclassification for people with events and non-events with clinically important categories of < 5%, 5–15%, and > 15%, which are thresholds used and suggested in perioperative prognostic literature as being relevant to patients and care providers [27, 28]. Risk classification tables were also generated, stratified by event and non-event status, as per the categories listed above. We calculated a Net Absolute Reclassification Index (NARI) using the same risk categories, estimated as the net number of correctly reclassified per 1000 patients.

Finally, we estimated the clinical usefulness of each model using decision curve analyses (DCA) [29, 30]. DCA graphically estimate the net benefit of each model across a range of thresholds for acting on prediction model results, with net benefit representing the weighted balance of identifying true positives against the harms of false positive results [30, 31]. The net benefit at the specific predicted risk threshold can be compared between models. Current perioperative guidelines [11] (though not specific to kidney failure populations) recommend that if an estimated risk for surgical inpatients is greater than 6.0% (based on the corresponding risk of 1 point on the RCRI), preoperative natriuretic peptides should be measured, and if elevated, postoperative troponins should be monitored to detect myocardial injury. Therefore, we focused on net benefit at this threshold for our DCA. As elective surgery settings are more likely to provide the necessary time for interventions to be applied, we performed a sensitivity analysis where the DCA was restricted to patients who received elective surgery.

## Results

### Cohort participants

**Fig. 1:**
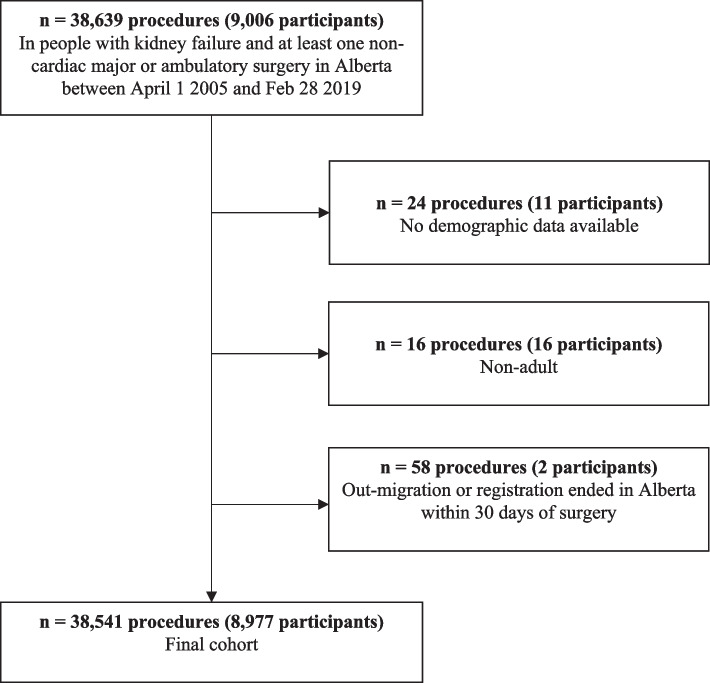
Cohort flow diagram. The number of patients that were included and excluded at each step of cohort formation are identified

We identified 38,541 surgeries performed in 8,997 participants (Fig. 1). The median number of procedures per person was 3 (IQR 1, 5). Most were performed in males (61%), with a median age of 64 years (IQR: 53, 73) (Table 1). The most common surgery type was AV fistula (26%), followed by vascular (21%), and skin and soft tissue surgery (14%). Seventy-four percent were performed in an ambulatory setting and in people receiving chronic hemodialysis (67%). The most common comorbidities were hypertension (96%), diabetes mellitus (66%), and heart failure (47%). The median (IQR) preoperative hemoglobin was 109 g/L (100,118) and albumin was 35 g/L 31, 32. Overall, there were 1,204 surgeries (3.1%) where death or a major cardiac event was recorded within 30 days of the surgery. The most common causes of death are summarized in Supplemental Table 6, and were most frequently cardiac, vascular, or a series of unspecified codes associated with diabetes or kidney failure.

**Table 1:** Cohort baseline characteristics

	No. (%)
**Total Surgeries**	38,541
**Female Sex**	14,949 (39)
**Age, years (median, IQR)**	64 (53, 73)
**Surgery Type**	
Arteriovenous fistula	9,889 (26)
Head & Neck	596 (2)
Intra-abdominal	1,737 (5)
Kidney Transplant	1,805 (5)
Musculoskeletal	2,083 (5)
Neurosurgery	306 (0.8)
Peritoneal Dialysis Catheter	4,014 (10)
Skin & Soft Tissue	5,336 (14)
Vascular	7,952 (21)
Low-risk Other	3,925 (10)
More than one	898 (2)
**Surgery Setting**	
Ambulatory	28,624 (74)
Major Elective	3,701 (10)
Major Urgent/Emergent	6,216 (16)
**Kidney Failure Type**	
Non-dialysis	9,781 (25)
Hemodialysis	25,706 (67)
Peritoneal Dialysis	3,054 (8)
**Comorbidities**	
Cancer	3,282 (9)
Cerebrovascular Disease	25,068 (65)
Chronic pulmonary disease	24,797 (64)
Dementia	2,399 (6)
Diabetes	25,424 (66)
Heart Failure	18,002 (47)
History of Myocardial Infarction	4,879 (13)
Hypertension	36,958 (96)
Liver disease	893 (2)
Obesity	9,186 (24)
Peripheral Vascular Disease	14,142 (37)
**Serum Hemoglobin, g/L (IQR)**	109 (100, 118)
Missing, imputed	78 (0.2)
**Serum Albumin, g/L (IQR)**	35 (31, 38)
Missing, imputed	5,589 (15)

### Model development, fit, and performance

Each model included all participants. Variable coefficients (odds ratios) and accompanying 95% confidence intervals are included in Table 2. Models 2 and 3 had similar estimates of AIC and BIC, indicating similar goodness of fit despite the inclusion of more variables (Table 2). The apparent c-statistics ranged from 0.785 (95% CI: 0.771, 0.800) for Model 1 to 0.818 (95% CI: 0.806, 0.831) for Model 3. The AUPRC was highest for Model 3 at 0.159. Apparent calibration slopes were estimated to be 1.00 (95% CI: 0.95, 1.05) for all models. Apparent performance was similar when restricted to only the first surgery per cohort participant (Supplementary Table 7).

**Table 2:** Variable coefficients and overall model performance for each risk prediction model

	**Model 1**	**Model 2**	**Model 3**
**Variable Name and Categories**	OR (95%CI)	OR (95%CI)	OR (95%CI)
*Age (per year above age 18)*	1.03 (1.02, 1.03)	1.02 (1.01, 1.03)	1.02 (1.01, 1.03)
*Female Sex*	0.98 (0.86, 1.12)	1.02 (0.89, 1.17)	1.00 (0.87, 1.15)
*Surgery Type*			
Intra-abdominal	Ref	Ref	Ref
Head and Neck	1.01 (0.65, 1.57)	1.04 (0.67, 1.61)	1.09 (0.70, 1.69)
Vascular	1.17 (0.91, 1.51)	1.07 (0.83, 1.38)	1.10 (0.85, 1.42)
Skin and Soft Tissue	0.99 (0.74, 1.32)	0.89 (0.66, 1.19)	0.82 (0.61, 1.11)
Neurosurgery	1.94 (1.16, 3.22)	2.04 (1.22, 3.41)	2.09 (1.25, 3.50)
Peritoneal Dialysis Catheter	0.52 (0.38, 0.72)	0.56 (0.41, 0.78)	0.54 (0.39, 0.75)
Arteriovenous Fistula	0.61 (0.47, 0.80)	0.60 (0.46, 0.79)	0.60 (0.46, 0.79)
Kidney Transplant	0.28 (0.19, 0.43)	0.37 (0.24, 0.56)	0.42 (0.28, 0.65)
Low risk – Other^a^	0.46 (0.32, 0.66)	0.44 (0.31, 0.64)	0.45 (0.31, 0.65)
More than one type	1.30 (0.97, 1.75)	1.23 (0.91, 1.66)	1.19 (0.88, 1.62)
Musculoskeletal	0.87 (0.67, 1.13)	0.84 (0.65, 1.10)	0.83 (0.64, 1.08)
*Surgery Setting*			
Ambulatory Surgery	Ref	Ref	Ref
Major Elective	3.37 (2.68, 4.24)	3.40 (2.69, 4.28)	3.36 (2.66, 4.25)
Major Urgent/Emergent	8.73 (7.55, 10.09)	7.69 (6.63, 8.92)	7.24 (6.23, 8.43)
*Kidney Failure Type*			
Hemodialysis	1.19 (1.00, 1.40)	1.06 (0.90, 1.27)	1.04 (0.87, 1.23)
Peritoneal Dialysis	1.35 (1.06, 1.70)	1.34 (1.05, 1.70)	1.13 (0.88, 1.44)
Non-dialysis	Ref	Ref	Ref
*Comorbidities*			
Cancer		1.28 (1.04, 1.57)	1.24 (1.01, 1.52)
Cerebrovascular disease		1.14 (0.99, 1.32)	1.12 (0.97, 1.29)
Chronic Pulmonary Disease		1.22 (1.06, 1.40)	1.23 (1.07, 1.42)
Dementia		1.20 (0.95, 1.50)	1.15 (0.91, 1.45)
Diabetes		1.22 (1.04, 1.43)	1.16 (0.99, 1.36)
Heart Failure		1.68 (1.44, 1.96)	1.64 (1.41, 1.91)
History of Myocardial Infarction		2.03 (1.72, 2.39)	2.02 (1.71, 2.38)
Hypertension		1.11 (0.70, 1.78)	1.14 (0.72, 1.81)
Liver disease		1.25 (0.87, 1.78)	1.15 (0.81, 1.63))
Obesity		0.83 (0.70, 0.99)	0.83 (0.69, 0.99)
Peripheral vascular disease		0.93 (0.80, 1.08)	0.87 (0.75, 1.01)
*Serum Albumin (per one unit change in g/L)*			0.95 (0.94, 0.96)
*Serum Hemoglobin (per one unit change in g/L)*			1.00 (1.00, 1.01)
*Constant (baseline odds)*	0.0041 (0.0028, 0.0060)	0.0030 (0.0016, 0.0054)	0.015 (0.0066, 0.035)
**Model Fit and Performance**			
*Number of surgeries included*	38,541	38,541	38,541
*Number of outcomes included*	1,204	1,204	1,204
*Akaike’s Information Criteria (AIC)*	9237	8996	8934
*Bayesian Information Criteria (BIC)*	9383	9236	9192
*Apparent Performance*			
C-statistic (95%CI)	0.785 (0.771, 0.800)	0.813 (0.800, 0.826)	0.818 (0.806, 0.831)
Area under precision recall curve	0.133	0.150	0.159
Calibration Slope (95%CI)	1.00 (0.95, 1.05)	1.00 (0.95, 1.05)	1.00 (0.95, 1.05)
*Optimism-adjusted Performance*			
C-statistic (95%CI)	0.783 (0.770, 0.797)	0.809 (0.798, 0.823)	0.814 (0.803, 0.826)
Expected to Observed Ratio (95%CI)	1.01 (0.97, 1.07)	1.01 (0.97, 1.07)	1.01 (0.97, 1.07)
Calibration intercept (95%CI)	0.00 (-0.06, 0.06)	0.00 (-0.06, 0.06)	0.00 (-0.06, 0.06)
Calibration slope (95%CI)	0.99 (0.94, 1.04)	0.98 (0.94, 1.03)	0.98 (0.94, 1.03)
*Comparison with simpler nested model*			
Net Reclassification Index (NRI)			
Total (95% CI), p-value	-	0.095 (0.063, 0.12), p < 0.00001^b^	0.016 (-0.004, 0.035), p = 0.12^c^
NARI (per 1000 patients)^d^	-	7.8	0.8
Integrated Discrimination Index (IDI)	-	0.016, *p* < 0.00001^b^	0.004, *p* < 0.00001^c^

*CI* confidence interval, *OR* odds ratio, *Ref* reference group for respective variable

^a^ Low-risk other surgery includes Anorectal, Breast, Lower Urologic and Gynecologic, Ophthalmology, Retroperitoneal, and Thoracic surgery types

^b^ Indicates that the comparison is between Model 2 and the more simple nested Model 1

^c^ Indicates that the comparison is between Model 3 and the more simple nested Model 2

^d^NARI is the Net Absolute Reclassification Index in number per 1000 patients, and is calculated as: (Proportion of reclassification for patients with events x event rate) + (proportion reclassification for patients without events x non-event rate) × 1000

**Fig. 2:**
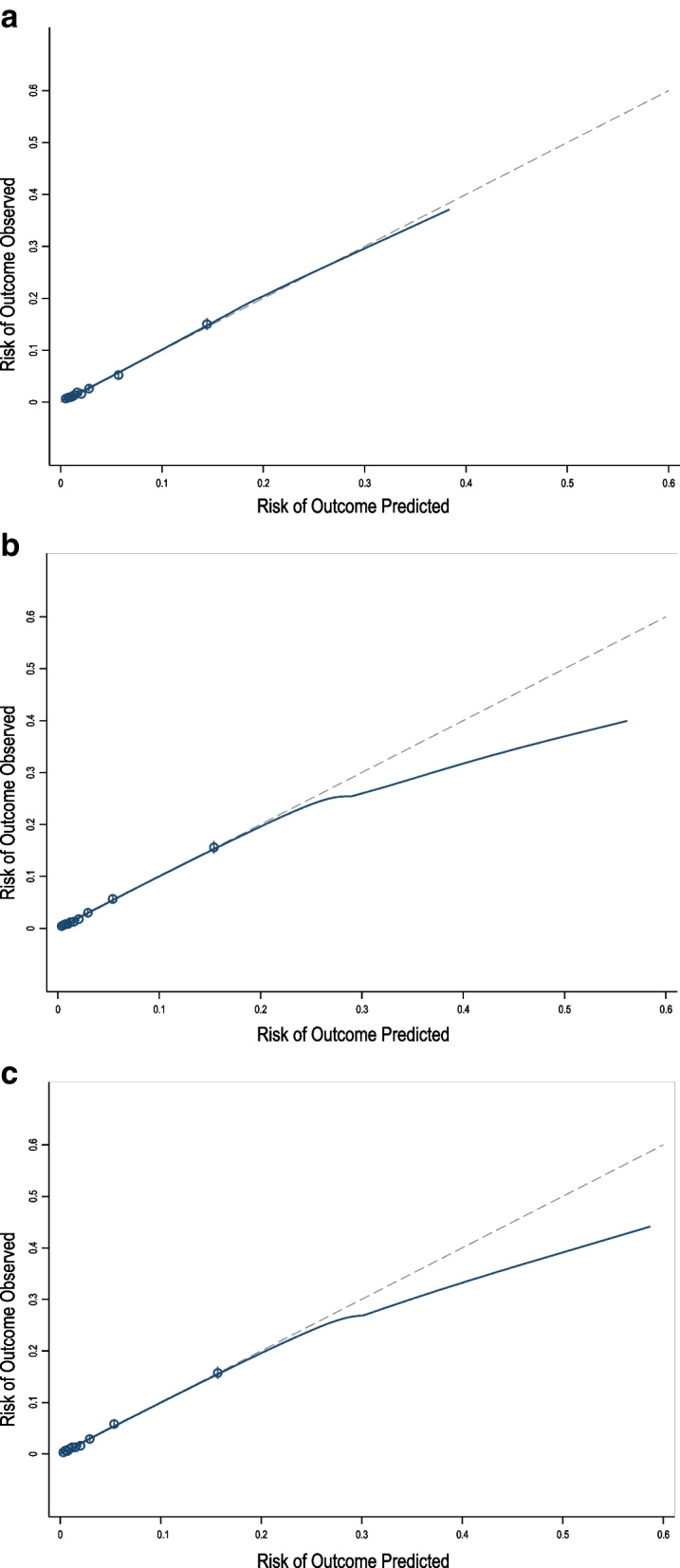
Calibration plots for each perioperative prediction model for people with kidney failure. The observed risk of 30-day cardiac or death events is plotted against the predicted risk in these calibration plots. The calibration plot for Model 1 is presented in Fig. 2a, Model 2 in Fig. 2b, Model 3 in Fig. 2c. The dashed line represents perfect calibration (with a slope of 1), and the solid line represents the lowess smoothed calibration curve. Each decile grouping of predicted risk is represented with an open circle along this calibration curve with accompanying 95% confidence intervals

Following internal validation, c-statistics derived through bootstrapping ranged from 0.783 (95% CI: 0.770, 0.797) for Model 1 to 0.814 (95% CI: 0.803, 0.826) for Model 3 (Table 2). The calibration slope point estimates ranged from 0.98 (Models 2 and 3) to 0.99 (Models 1) and calibration intercepts were near 0 for all models. Examination of the calibration plot for Models 1 showed excellent calibration across all predicted risks (Fig. 2a), and for models 2 and 3 predictions were well calibrated except for overestimation at the highest predicted risks (above 30%) (Fig. 2b-c). The addition of variables from Model 1 to Model 2 led to statistically significant and improved reclassification of individuals, with a categorical NRI of 0.1 (95%CI: 0.06, 0.12; *p* < 0.00001) and a NARI of 7.8 per 1000 patients. The addition of hemoglobin and albumin to Model 3 resulted in a small but not significant improvement in reclassification when compared with Model 2, with a categorical NRI of 0.02 (95% CI: -0.004, 0.04; *p* = 0.12) and a NARI of 0.8 per 1000 patients. The estimated IDI’s also significantly improved when comparing Model 2 with Model 1, and Model 3 with Model 2. Improvement in classification, stratified by those with and without events, is presented in Supplementary Table 8.

**Fig. 3:**
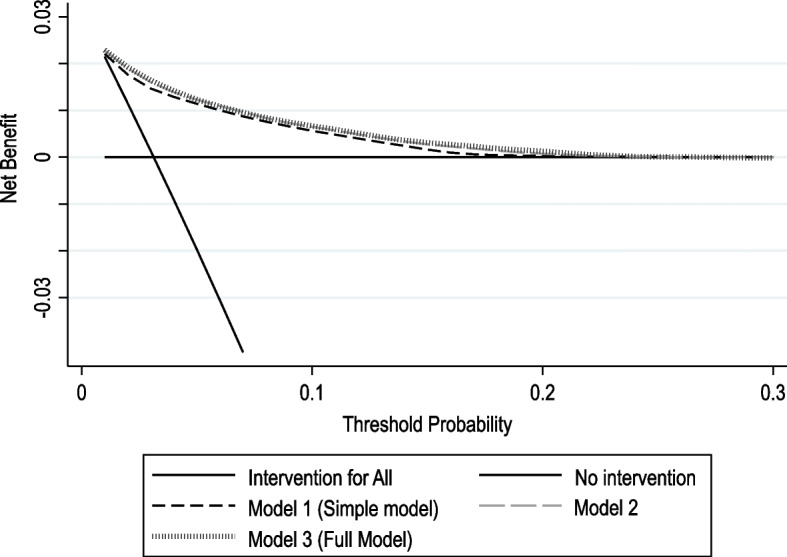
Decision curve analysis comparing the clinical usefulness for the three risk prediction models versus strategies where perioperative interventions were applied in all or no participants

We estimated the potential clinical usefulness of all three models using DCA. All three models had similar positive net benefit (at the prespecified 6.0% predicted risk threshold) over strategies where all or none of the participants received a perioperative cardiac monitoring intervention, with all models having net benefit if used to guide perioperative cardiac monitoring based on predicted risk thresholds from 0 and 20% (Fig. 3). When we applied the models only to surgeries performed in ambulatory or inpatient elective settings, we found similar net benefit (Supplementary Fig. 1).

## Discussion

In this study, we derived and internally validated three multivariable risk prediction models to estimate the risk of major postoperative events for people with kidney failure. Our models included sets of surgical, demographic, comorbidity, and laboratory predictors that are prevalent and frequently measured in the kidney failure population and common perioperative clinical scenarios. All three models performed well and were estimated to be clinically useful to inform perioperative decision making when examined using DCA. The models that included comorbidities and laboratory variables had the best discrimination and resulted in improvement in reclassification of patients with and without events into higher or lower risk categories when compared with the simplest model, suggesting that this full model (Model 3) is preferred for perioperative risk assessment of patients with kidney failure when comorbidity and laboratory data are available.

This work has important implications for the care of people with kidney failure since people with kidney failure frequently require surgery, and have a high risk of adverse postoperative outcomes [6]. These models address many of the limitations of current perioperative risk prediction tools for people with kidney failure by including predictors that are relevant and unique to people with kidney failure. Though comorbidities are often included in postoperative outcome models, we found it interesting that inclusion of preoperative hemoglobin and albumin improved model performance. Preoperative anemia is well known to be associated with postoperative major outcomes such as death [32], and preoperative hypoalbuminemia as a marker of frailty or volume overload has been identified as a risk factor for postoperative death and many cardiac outcomes [33]. When we compare our models to those commonly used in the perioperative realm, there are several notable differences. North American preoperative risk stratification guidelines recommend the use of tools such as the Revised Cardiac Risk Index (RCRI) [34], the National Surgical Quality Improvement Program (NSQIP) American College of Surgery (ACS) tool [35, 36], and the NSQIP Myocardial Infarction or Cardiac Arrest (MICA) tool [37]. Many other tools have also been developed for the prediction of death and/or major cardiac events after surgery [10]. The ACS tool includes a variable for preoperative dialysis but does not discriminate between people receiving maintenance dialysis from those with acute kidney injury (AKI). The widely used RCRI and MICA include dichotomized variables for a serum creatinine above 177 μmol/L and 133 μmol/L, respectively, which does not discriminate risk between moderate CKD and kidney failure. As the nephrology community has used eGFR coupled with albuminuria to categorize kidney function rather than serum creatinine for some time [38], there is work underway to update the RCRI to include eGFR in place of serum creatinine [28]. However, the validity of this updated RCRI for people with kidney failure receiving dialysis is unclear. We have recently found that the performance of the RCRI in a kidney failure cohort was poor, with significant overestimation of risk, and modest improvement with re-estimation of model coefficients [12]. Comparison of the original and updated RCRI, MICA, and ACS tools with our models externally in independent cohorts of people with kidney failure may identify the best models to adopt within perioperative settings for patients with kidney failure.

After evaluating model performance, we estimated the clinical usefulness of each model using DCA. With this technique, the net benefit of risk stratification represents the net proportion of true positives that would be identified, balancing the risk of false positive identification at each probability threshold [30]. In the setting of perioperative care, risk prediction models could be used to guide planned admission to hospital following elective surgery, postoperative cardiac monitoring with electrocardiograms and systematic troponin measurement, optimization of risk factors for postoperative events (such as anemia), tailoring anesthetic strategies, or consideration of specific medications that may reduce the risk of postoperative events. As our cohort included only people that had surgery performed, the application of our DCA is most relevant to decisions regarding interventions aimed at reducing perioperative risk and not for determining surgical eligibility. Recent guidelines recommend that a perioperative cardiac risk above approximately 6.0% should be used to guide perioperative cardiac monitoring, which is a threshold at which decision making based on our models would have net benefit. Future work should examine whether these thresholds determined for the general population are appropriate to this patient population with kidney failure, and whether other thresholds to inform alternative perioperative interventions are priorities. It is possible that different thresholds would be prioritized by people with kidney failure and their care providers for other perioperative decisions (e.g. admission to hospital).

There are limitations to this study. First, though we used validated case definitions for comorbidities and our outcomes, these algorithms may be specific but not very sensitive (especially for non-fatal outcomes), and may lead to underestimation of overall risk. Preoperative natriuretic peptides, although helpful for prognosticating patients at risk of postoperative events in the general population [27], are difficult to interpret in the setting of kidney failure [39], and administrative data sources cannot determine whether such tests were ordered for perioperative indications rather than evaluation of heart failure or dyspnea. Further, some risk factors for intraoperative hypotension such as mode of anesthesia delivery could not be ascertained from our administrative data sources. Finally, even within some predictors with many categories, such as surgery type, each category still will have significant outcome risk variability within it which will not be accounted for in the model estimates. As an example, postoperative mortality following a subarachnoid hemorrhage surgery may in reality be much higher than an urgent brain tumor resection. Though our model calibration was excellent overall, calibration in each predictor category is a separate consideration which may have impact on interpretability of each risk estimate for each patient context.

There are several future directions for this work. First, these models need to be externally validated either using existing data to establish their generalizability before they could be implemented and evaluated further. Ideally, we could validate our models in a prospective perioperative cohort of people with kidney failure, which could also allow for the incorporation of other variables not yet feasible in a retrospective administrative data cohort and could improve model performance. Formal comparison of our models with perioperative models not specific to people with kidney failure using DCA could be completed, with kidney failure specific decision thresholds, as mentioned above. Finally, before widespread use and implementation, evaluation of perioperative strategies informed by our risk prediction models could be completed in interventional (ideally randomized) study designs.

In summary, we derived and internally validated three well-performing and clinically useful risk prediction models to predict the risk of cardiac events and death within 30 days of surgery for people with kidney failure. Once externally validated, these models may inform perioperative shared decision making and guide the use of perioperative interventions for this important group with frequent surgeries and higher risk of poor postoperative outcomes.

## Supplementary Information

**Additional file 1: Supplementary Table 1.** Transparent reporting of a multivariable prediction model for individual prognosis or diagnosis (TRIPOD) Checklist for Prediction model development. **Supplementary Table 2.** Algorithms of ICD-9 and 10 codes used to define components of our composite outcome. **Supplementary Table 3.** Candidate Predictor definition along with source of data and ICD-9/10 algorithms if applicable. **Supplementary Table 4.** Surgical Categories by Canadian Classification of Health Intervention (CCI) codes. **Supplementary Table 5. **Estimated Sample Size Calculations using ‘pmsampsize’ in Stata software v17.0 and as suggested by Riley et al (2020). **Supplementary Table 6. **Top causes of death for those that died within 30 days of surgery, with associated ICD-10 codes. **Supplementary Table 7.** Performance of models evaluated in cohort with only first surgery per participant. **Supplementary Table 8.** Event and non-eventReclassification Tables between models, stratified by clinically important probability categories. **Supplementary Figure 1.** Decision Curve Analysis to estimate the net benefit of use of perioperative risk prediction models in ambulatory or inpatient elective surgery (sensitivity analysis).

## Data Availability

This study is based in part on data provided by Alberta Health and Alberta Health Services, and is not publicly available for sharing. The interpretation and conclusions contained herein are those of the researchers and do not necessarily represent the views of the Government of Alberta or Alberta Health Services. Neither the Government of Alberta, Alberta Health or Alberta Health Services express any opinion in relation to this study. We are not able to make our dataset available to other researchers due to our contractual arrangements with the provincial health ministry (Alberta Health), who is the data custodian. Researchers may make requests to obtain a similar dataset at https://www.alberta.ca/health-research.aspx.
