# Supplementary material for: Prediction of major postoperative events after non-cardiac surgery for people with kidney failure: derivation and internal validation of risk models
Source: BMC Nephrol. 2023 Mar 10;24:49. doi: 10.1186/s12882-023-03093-6 (PMC9999551; doi:10.1186/s12882-023-03093-6)
Supplement: Supplementary file 1 — Additional file 1: Supplementary Table 1. Transparent reporting of a multivariable prediction model for individual prognosis or diagnosis (TRIPOD) Checklist for Prediction model development. Supplementary Table 2. Algorithms of ICD-9 and 10 codes used to define components of our composite outcome. Supplementary Table 3. Candidate Predictor definition along with source of data and ICD-9/10 algorithms if applicable. Supplementary Table 4. Surgical Categories by Canadian Classification of Health Intervention (CCI) codes. Supplementary Table 5. Estimated Sample Size Calculations using ‘pmsampsize’ in Stata software v17.0 and as suggested by Riley et al (2020). Supplementary Table 6. Top causes of death for those that died within 30 days of surgery, with associated ICD-10 codes. Supplementary Table 7. Performance of models evaluated in cohort with only first surgery per participant. Supplementary Table 8. Event and non-eventReclassification Tables between models, stratified by clinically important probability categories. Supplementary Figure 1. Decision Curve Analysis to estimate the net benefit of use of perioperative risk prediction models in ambulatory or inpatient elective surgery (sensitivity analysis). [file 12882_2023_3093_MOESM1_ESM.docx]

**Prediction of major postoperative events after non-cardiac surgery for people with kidney failure: Derivation and internal validation of risk models**

Harrison TG et al.

**Supplementary Material**

**Supplementary Table 1.** Transparent reporting of a multivariable prediction model for individual prognosis or diagnosis (TRIPOD) Checklist for Prediction model development

**Supplementary Table 2.** Algorithms of ICD-9 and 10 codes used to define components of our composite outcome

**Supplementary Table 3.** Candidate Predictor definition along with source of data and ICD-9/10 algorithms if applicable

**Supplementary Table 4.** Surgical Categories by Canadian Classification of Health Intervention (CCI) codes

**Supplementary Table 5.** Estimated Sample Size Calculations using ‘pmsampsize’ in Stata software v17.0 and as suggested by Riley et al (2020)

**Supplementary Table 6.** Top causes of death for those that died within 30 days of surgery, with associated ICD-10 codes

**Supplementary Table 7.** Performance of models evaluated in cohort with only first surgery per participant

**Supplementary Table 8.** Event and non-event Reclassification Tables between models, stratified by clinically important probability categories

**Supplementary Figure 1.** Decision Curve Analysis to estimate the net benefit of use of perioperative risk prediction models in ambulatory or inpatient elective surgery (sensitivity analysis)

**Supplementary Table 1.** Transparent reporting of a multivariable prediction model for individual prognosis or diagnosis (TRIPOD) Checklist for Prediction model development

| **Section/Topic** | **Ite** | **Checklist Item** | **Page** |
| --- | --- | --- | --- |
| **Title and abstract** | | | |
| Title | 1 | Identify the study as developing and/or validating a multivariable prediction model, the target population, and the outcome to be predicted. | 1 |
| Abstract | 2 | Provide a summary of objectives, study design, setting, participants, sample size, predictors, outcome, statistical analysis, results, and conclusions. | 4,5 |
| **Introduction** | | | |
| Background and objectives | 3a | Explain the medical context (including whether diagnostic or prognostic) and rationale for developing or validating the multivariable prediction model, including references to existing models. | 7 |
|  | 3b | Specify the objectives, including whether the study describes the development or validation of the model or both. | 7 |
| **Methods** | | | |
| Source of data | 4a | Describe the study design or source of data (e.g., randomized trial, cohort, or registry data), separately for the development and validation data sets, if applicable. | 7,8 |
|  | 4b | Specify the key study dates, including start of accrual; end of accrual; and, if applicable, end of follow-up. | 8 |
| Participants | 5a | Specify key elements of the study setting (e.g., primary care, secondary care, general population) including number and location of centres. | 8 |
|  | 5b | Describe eligibility criteria for participants. | 8 |
|  | 5c | Give details of treatments received, if relevant. | N/A |
| Outcome | 6a | Clearly define the outcome that is predicted by the prediction model, including how and when assessed. | 8,9 |
|  | 6b | Report any actions to blind assessment of the outcome to be predicted. | N/A |
| Predictors | 7a | Clearly define all predictors used in developing or validating the multivariable prediction model, including how and when they were measured. | 9 |
|  | 7b | Report any actions to blind assessment of predictors for the outcome and other predictors. | N/A |
| Sample size | 8 | Explain how the study size was arrived at. | 9,10 |
| Missing data | 9 | Describe how missing data were handled (e.g., complete-case analysis, single imputation, multiple imputation) with details of any imputation method. | 9 |
| Statistical analysis methods | 10a | Describe how predictors were handled in the analyses. | 10 |
|  | 10b | Specify type of model, all model-building procedures (including any predictor selection), and method for internal validation. | 10,11 |
|  | 10d | Specify all measures used to assess model performance and, if relevant, to compare multiple models. | 10,11 |
| Risk groups | 11 | Provide details on how risk groups were created, if done. | N/A |
| **Results** | | | |
| Participants | 13a | Describe the flow of participants through the study, including the number of participants with and without the outcome and, if applicable, a summary of the follow-up time. A diagram may be helpful. | 12, Fig 1 |
|  | 13b | Describe the characteristics of the participants (basic demographics, clinical features, available predictors), including the number of participants with missing data for predictors and outcome. | 12, Tab 1 |
| Model development | 14a | Specify the number of participants and outcome events in each analysis. | 12 |
|  | 14b | If done, report the unadjusted association between each candidate predictor and outcome. | N/A |
| Model specification | 15a | Present the full prediction model to allow predictions for individuals (i.e., all regression coefficients, and model intercept or baseline survival at a given time point). | Tab 2 |
|  | 15b | Explain how to the use the prediction model. | 12,13 |
| Model performance | 16 | Report performance measures (with CIs) for the prediction model. | 12,13, Tab 2 |
| **Discussion** | | | |
| Limitations | 18 | Discuss any limitations of the study (such as nonrepresentative sample, few events per predictor, missing data). | 15 |
| Interpretation | 19b | Give an overall interpretation of the results, considering objectives, limitations, and results from similar studies, and other relevant evidence. | 13,14 |
| Implications | 20 | Discuss the potential clinical use of the model and implications for future research. | 13-16 |
| **Other information** | | | |
| Supplementary information | 21 | Provide information about the availability of supplementary resources, such as study protocol, Web calculator, and data sets. | 6 |
| Funding | 22 | Give the source of funding and the role of the funders for the present study. | 6 |

*Reference: Collins GS, Reitsma JB, Altman DG, Moons KG. Transparent reporting of a multivariable prediction model for individual prognosis or diagnosis (TRIPOD): The TRIPOD statement. Ann Intern Med. 2015;162(1):55-63. PMID: [25560714](http://www.ncbi.nlm.nih.gov/pubmed/25560714)

**Supplementary Table 2.** Algorithms of ICD-9 and 10 codes used to define components of our composite outcome

| **Component Outcomes** | **ICD-9-CM diagnostic codes** | **ICD-10-CA diagnostic codes** | **Validation or Summary Studies** | **Data Source** |
| --- | --- | --- | --- | --- |
| Death | Any cause of death from Vital Statistics file | Any cause of death from Vital Statistics file | N/A | Vital Statistics |
| Acute Myocardial Infarction | 410 | I21 I22 | ^1, 2^ | Hospitalization data file |
| Non-fatal cardiac arrest or ventricular arrythmia | 427.1, 427.4, 427.41, 427.42, 427.5, 427.9, 798, 798.1, 798.2 | I472 I4901 I4902 I469 I499 R99 (converted from ICD-9) | ^3^ | Hospitalization data file |

ICD, International Statistical Classification of Diseases (9^th^ and 10^th^ revision).

**Supplementary Table 3.** Candidate Predictor definition along with source of data and ICD-9/10 algorithms if applicable

| **Data Element** | **Data Element Source** | **Data element Description** |
| --- | --- | --- |
| ***Demographic Variables*** | | |
| Age | Alberta Health Registry | Continuous in years, calculated as index date minus date of birth, centred at age 18. |
| Sex | Alberta Health Registry | Dichotomous Male and Female |
| ***Surgical Variables*** | | |
| Category of surgery | Hospitalization dataset (CCI codes) | Categorized into 16 surgical groups per CCI codes as outlined in Supplementary Table 4. |
| Surgery setting | Ambulatory Care dataset, Hospitalization dataset (SEPI_ADMIT_CAT code for admission type) | Categorized into three categories: Ambulatory surgery based on surgery location/no planned admission; Major elective based on planned hospital admission with inpatient surgery; Major Urgent/Emergent based on unplanned hospital admission with inpatient surgery. |
| ***Kidney Failure Variables*** | | |
| Kidney Failure Type | Renal registry records | Coded as hemodialysis, peritoneal dialysis, or non-dialysis. |
| ***Comorbidities (all defined with unrestricted lookback)^4^*** | | |
| Cancer (any) | Hospitalization (ICD-10-CA) and Physician claims (Enhanced ICD-9-CM) | ICD-10: C00.x–C26.x, C30.x–C34.x, C37.x–  C41.x, C43.x, C45.x–C58.x, C60.x–C76.x, C81.x–C85.x, C88.x, C90.x–C97.x; ICD-9: 140.x–172.x, 174.x–195.8, 200.x–208.x, 238.6  ICD-10: C77.x-C80.x; ICD-9: 196.x-199.x |
| Cerebrovascular disease | Hospitalization (ICD-10-CA) and Physician claims (Enhanced ICD-9-CM) | ICD-10: G45.x, G46.x, H34.0, 160.x-169.x; ICD-9: 362.34, 430.x-438.x |
| Chronic Pulmonary Disease | Hospitalization (ICD-10-CA) and Physician claims (Enhanced ICD-9-CM) | ICD-10: I27.8, I27.9, J40.x–J47.x, J60.x–J67.x,  J68.4, J70.1, J70.3; ICD-9: 416.8, 416.9, 490.x–505.x, 506.4, 508.1, 508.8 |
| Dementia | Hospitalization (ICD-10-CA) and Physician claims (Enhanced ICD-9-CM) | ICD-10: F00.x–F03.x, F05.1, G30.x, G31.1 290.x; ICD-9: 294.1, 331.2 |
| Diabetes | Hospitalization (ICD-10-CA) and Physician claims (Enhanced ICD-9-CM) | ICD-10: E10-14; ICD-9: 250 |
| Heart Failure | Hospitalization (ICD-10-CA) and Physician claims (Enhanced ICD-9-CM) | ICD-10: I09.9, I11.0, I13.0, I13.2, I25.5, I42.0, I42.5-I42.9, I43.x, I50.x, P29.0; ICD-9: 398.91, 402.01, 402.11, 402.91, 404.01, 404.03, 404.11, 404.13, 404.91, 404.93, 425.4–425.9, 428.x |
| History of Myocardial Infarction | Hospitalization (ICD-10-CA) and Physician claims (Enhanced ICD-9-CM) | ICD-10: I21.x, I22.x, I25.2; ICD-9:410.x, 412.x |
| Hypertension | Hospitalization (ICD-10-CA) and Physician claims (Enhanced ICD-9-CM) | ICD-10: I10-I13, I15  ICD-9: 401-405 |
| Liver disease (mild and moderate/severe) | Hospitalization (ICD-10-CA) and Physician claims (Enhanced ICD-9-CM) | ICD-10: B18.x, K70.0–K70.3, K70.9,  K71.3–K71.5, K71.7, K73.x, K74.x,  K76.0, K76.2–K76.4, K76.8, K76.9,  Z94.4; ICD-9: 070.22, 070.23, 070.32, 070.33,  070.44, 070.54, 070.6, 070.9,  570.x, 571.x, 573.3, 573.4,  573.8, 573.9, V42.7  ICD-10: I85.0, I85.9, I86.4, I98.2, K70.4,  K71.1, K72.1, K72.9, K76.5, K76.6,  K76.7; ICD-9: 456.0–456.2, 572.2–572.8 |
| Obesity^1^ | Physician claims (Enhanced ICD-9-CM) | Procedural modifier codes for billing: BMI, BMIABD, BMIANE, BMIANT, BMISRG, BMI2AN, BMIPRO. ^5^ |
| Peripheral Vascular disease | Hospitalization (ICD-10-CA) and Physician claims (Enhanced ICD-9-CM) | ICD-10: I70.x, I71.x, I73.1, I73.8, I73.9, I77.1,  I79.0, I79.2, K55.1, K55.8, K55.9, Z95.8, Z95.9; ICD-9: 093.0, 437.3, 440.x, 441.x, 443.1–443.9, 47.1, 557.1, 557.9, V43.4 |
| ***Laboratory investigations*** | | |
| Hemoglobin (g/L) | Alberta Laboratory data | Most recent preoperative outpatient hemoglobin that was drawn prior to the procedure, i.e. date of hemoglobin measure minus admission date is ≥1 day, from outpatient lab, and within one year before the procedure itself. |
| Albumin (g/L) | Alberta Laboratory data | Most recent preoperative outpatient albumin drawn prior to the procedure, i.e. date of albumin measure minus admission date is ≥1 day, from outpatient lab, and within one year before the procedure itself. |

^1^Obesity definition based on algorithm of physician claims for High Body Mass Index >35 kg/m2 before January 1 2017 and >40 after.

**Supplementary Table 4.** Surgical Categories by Canadian Classification of Health Intervention (CCI) codes

| **Surgery Type** | **Section, Group, Intervention Components Codes based on Canadian Classification of Interventions (CCI)** |
| --- | --- |
| Anorectal | 1NQ56DA 1NQ56LA 1NQ59DAAD 1NQ59DAAG 1NQ59DAGX 1NQ59HAX7 1NQ59LAAD 1NQ59LAAG 1NQ59LAGX 1NQ72DA 1NQ72LA 1NQ72PB 1NQ74DW 1NQ74ED 1NQ74EJ 1NQ74PC 1NQ74PD 1NQ74PE 1NQ74SS 1NQ74TV 1NQ74VT 1NQ80 1NQ84 1NQ86 1NQ87CA 1NQ87DA 1NQ87DF 1NQ87DX 1NQ87LA 1NQ87PB 1NQ87PF 1NQ87RD 1NQ87TF 1NQ89 1NQ90 1NT53LADV 1NT53LAPM 1NT56LA 1NT72 1NT80 1NT84 1NT86 1NT87 |
| Arteriovenous fistula | 1JM50 1JM51 1JM57 1JM58 1JM76 1JM80 1JM82 1JM87 1KY76LA 1KY76LASJ 1KY76LAXXA 1KY76LAXXL 1KY76LAXXN 1KY80 1KY76 |
| Breast | 1YK50 1YK58 1YK80 1YK83 1YK84 1YK87 1YK89 1YK90 1YL87 1YL89 1YM58 1YM74 1YM78 1YM79 1YM80 1YM87 1YM88 1YM89 1YM90 1YM91 1YM92 |
| Head and Neck | 1DA56LA 1DA58 1DA59HAT9 1DA59JAGX 1DA80 1DA82 1DA83 1DA84 1DA87 1DA89 1DA91 1DE56LA 1DE59JAGX 1DE80 1DE84 1DE86 1DE87 1DE91 1DF53 1DF58 1DF71JA 1DF72LA 1DF80 1DF85 1DF87 1DF89 1DG 1DJ53 1DJ80 1DK59LAGX 1DK80 1DK85 1DK87 1DK91 1DE53 1DL59LAGX 1DL80 1DL87 1DL89 1DL91 1DM53 1DN76QRQB 1DP53 1DR57 1DR59LAKD 1DR59QRAZ 1DR59QRKD 1DR59QRX7 1DR72 1DR80 1DR89 1DR91 1DZ70 1DZ94 1EA56 1EA58 1EA72 1EA73 1EA74 1EA80 1EA87 1EA92 1EB73LA 1EB74 1EB80 1EB87 1EC 1ED56 1ED73 1ED74 1ED79 1ED80 1ED83 1ED87 1ED91 1EE56 1EE58 1EE71 1EE73 1EE78 1EE79 1EE80 1EE83 1EE87 1EE91 1EF73 1EF74 1EF80 1EG 1EH 1EJ87 1EJ89 1EL53 1EL57LA 1EL72DA 1EL72LA 1EL74 1EL80 1EL83 1EM53 1EM73LA 1EM74 1EM80 1EM86 1EM87 1EN53 1EN73LA 1EN74 1EN80 1EN87 1EN91 1EP58 1EP72 1EP80 1EP87 1EQ56LA 1EQ59 1EQ70 1EQ87 1EQ94 1ES58 1ES80 1ES87 1ET56LA 1ET57LA 1ET59LAAD 1ET59LAAG 1ET59LAGX 1ET72LA 1ET73 1ET80 1ET82 1ET86 1ET87 1ET89 1EU87 1EU89 1EV87 1EW80 1EW86 1EW87 1EW91 1EX59 1EX80 1EX87 1EY87 1EY91 1FA53 1FA56LA 1FA84 1FA87 1FA91 1FB53 1FB80 1FB86 1FB87 1FB91 1FC56LA 1FC80LAXXB 1FC80LAXXE 1FC87 1FG56LA 1FG58 1FG59JAGX 1FG72 1FG80 1FG87 1FH56LA 1FH59JAGX 1FH78 1FH80 1FH87 1FJ56LA 1FJ59JAGX 1FJ72 1FJ74 1FJ80 1FJ87 1FJ91 1FK94 1FL51 1FL57 1FL80 1FL87 1FL89 1FM50 1FM51 1FM57 1FM80 1FM83 1FM87 1FM89 1FM91 1FN51 1FN57 1FN59 1FN80 1FN83 1FN87 1FN89 1FQ56LA 1FQ59HAAW 1FQ78 1FQ80 1FQ87 1FQ89 1FR56LA 1FR59JAGX 1FR87 1FR89 1FU71 1FU87 1FU89 1FU91 1FV83 1FV87 1FV89 1FX56LA 1FX80 1FX86 1FX87 1FX91 1GA74 1GA80 1GA83 1GA87 1GA89 1GB 1GC50 1GC59 1GD53 1GD74 1GD83 1GD87 1GD89 1GE50 1GE56LA 1GE80 1GE87 1GE89 1GE91 1GH71 1GH84 1GJ50LA 1GJ50LANR 1GJ53LAPM 1GJ56LA 1GJ77 1GJ80 1GJ82 1GJ85 1GJ86 1GJ87 1GK52LA 1GK59LAGX 1GK74LA 1GK80 1GK83 1GK87 1GK89 1MB87LA 1MC87 1MC89 1MC91 1ML59 1ML87 |
| Intra-abdominal | 1MG87 1MG89 1MJ87 1MJ89 1MJ91 1MP50 1MP51 1MP59 1MP76 1MP80 1MP87 1NF80DAXXE 1NF80DAXXN 1NF80LA 1NF80LAXXE 1NF80LAXXN 1NF82 1NF84 1NF86 1NF87 1NF89 1NF90 1NF91 1NF92 1NK53DATS 1NK53LAQB 1NK53LATS 1NK56DA 1NK56LA 1NK58 1NK74 1NK76 1NK77 1NK80 1NK82 1NK84 1NK85 1NK87DA 1NK87DN 1NK87DP 1NK87DX 1NK87DY 1NK87LA 1NK87RE 1NK87RF 1NK87TF 1NK87TG 1NM56DA 1NM56LA 1NM58 1NM74 1NM76 1NM77 1NM80 1NM82 1NM87 1NM89 1NM91 1NP58 1NP72 1NP73LA 1NP85 1NP86 1NV89 1OA53 1OA58 1OA59DAGX 1OA59LAGX 1OA74 1OA85 1OA87 1OB59DAGX 1OB59LAGX 1OB74 1OB83 1OB85 1OB87 1OB89 1OD57 1OD76 1OD80 1OD86 1OD89 1OE57DAAG 1OE57DAAM 1OE57DAAS 1OE57DAAZ 1OE57DABD 1OE57DAGX 1OE57HAAG 1OE57HAAM 1OE57HAAS 1OE57HAAZ 1OE57HABD 1OE57HAGX 1OE57LAAG 1OE57LAAM 1OE57LAAS 1OE57LAAZ 1OE57LABD 1OE57LAGX 1OE59KQ 1OE76 1OE80 1OE84 1OE86 1OE87 1OE89 1OJ53 1OJ56 1OJ76 1OJ83 1OJ85 1OJ87 1OJ89 1OK58 1OK85 1OK87 1OK89 1OK91 1OT56 1OT58 1OT70 1OT72 1OT80 1OT87 1OT91 1OW12 1OW80DA 1OW80LA 1OW87 1OW89 1OZ94LA |
| Kidney Transplant | 1PC85 |
| Lower Urologic/Gynecologic | 1MH87 1MH89 1PQ53LAPZ 1PQ56DA 1PQ56LA 1PQ56QY 1PQ57LAAM 1PQ57LAGX 1PQ59FLAD 1PQ59FLAG 1PQ59FLAS 1PQ59FLAZ 1PQ59FLGX 1PQ59LAAZ 1PQ59LAGX 1PQ72 1PQ77 1PQ78 1PQ80 1PQ82 1PQ86 1PQ87 1PQ89 1PV57LAGX 1PV59LAGX 1PV80 1PZ94DA 1PZ94HA 1PZ94LA 1QD72 1QD89 1QE14JAXXK 1QE14JAXXL 1QE14JAXXN 1QE14JAXXP 1QE53 1QE56 1QE58 1QE59LAAD 1QE59LAAG 1QE59LAGX 1QE59LAX7 1QE72 1QE76 1QE80 1QE82 1QE84 1QE87 1QE89 1QG14JAXXK 1QG14JAXXL 1QG14JAXXN 1QG14JAXXP 1QG53 1QG56 1QG59LAAD 1QG59LAAG 1QG59LAGX 1QG59LAX7 1QG78 1QG80 1QG87 1QG89 1QH80 1QH87 1QJ53 1QJ58 1QJ80 1QJ87 1QJ89LA 1QM56 1QM58 1QM74 1QM80 1QM87 1QM89 1QM91 1QN 1QP51 1QP52 1QP72 1QP73 1QP87 1QQ87 1QQ89 1QT87PB 1QT87PK 1QT87PNGX 1QT87QZ 1QT87QZAG 1QT91 1QZ89 1QZ94DA 1QZ94HA 1QZ94LA 1RB56LA 1RB57DA 1RB57LA 1RB58 1RB59 1RB74 1RB80 1RB83 1RB85 1RB87 1RB89 1RD72 1RD89 1RF50DABJ 1RF50DAGX 1RF50DAKR 1RF50DANR 1RF50LABJ 1RF50LAGX 1RF50LAKR 1RF50LANR 1RF51 1RF56 1RF59DAAG 1RF59DAGX 1RF59LAAG 1RF59LAGX 1RF72 1RF74 1RF80 1RF87 1RF89 1RM56DA 1RM56LA 1RM59DAGX 1RM59LAGX 1RM72CAGX 1RM72DAGX 1RM72LAGX 1RM74 1RM80DA 1RM80LA 1RM80LAXXA 1RM80LAXXE 1RM80LAXXF 1RM80LAXXN 1RM80LAXXQ 1RM87DAAG 1RM87DAAK 1RM87DAGX 1RM87LAAK 1RM87LAGX 1RM89 1RM91 1RN74LA 1RN80DA 1RN80LA 1RN80LAFA 1RN80LAXXA 1RN80LAXXE 1RN89LA 1RN89LANRA 1RN89LANRE 1RN89LAXXA 1RN89LAXXE 1RN89LAXXQ 1RS59DAGX 1RS74 1RS80CAXXA 1RS80CAXXB 1RS80CAXXE 1RS80CAXXG 1RS80CAXXN 1RS80CAXXQ 1RS80DA 1RS80LA 1RS80LAXXA 1RS80LAXXB 1RS80LAXXE 1RS80LAXXG 1RS80LAXXN 1RS80LAXXQ 1RS84 1RS86 1RS87 1RS89 1RW56LA 1RW59 1RW72 1RW80 1RW84 1RW87 1RW88 1RW91 1RW92 1RY14JAXXK 1RY14JAXXN 1RY14JAXXP 1RY56LA 1RY59JAGX 1RY80 1RY87 1RZ94DA 1RZ94LA |
| Musculoskeletal (MSK) | 1VZ94LA 1VZ70LA 1VS80 1VS72 1VS58 1VR80 1VR72 1VR58 1VR57 1VQ93 1VQ91 1VQ87 1VQ83 1VQ82 1VQ80 1VQ79 1VQ74 1VQ73LA 1VQ58 1VP89 1VP87 1VP80 1VP74 1VP73LA 1VP72 1VP53 1VN 1VM 1VL 1VK89 1VK87 1VK80 1VG93 1VG87 1VG83 1VG80 1VG75 1VG74 1VG73LA 1VG72LA 1VG72DA 1VG58DA 1VG57 1VG53 1VE80 1VE72 1VE58 1VD87 1VD80 1VD72 1VD58 1VD57 1VC93 1VC91 1VC87 1VC83 1VC82 1VC80 1VC79 1VC74 1VC73LA 1VC58 1VA93 1VA87 1VA83 1VA80 1VA75 1VA74 1VA73LA 1VA72LA 1VA72DA 1VA58DA 1VA57 1VA53 1UV80 1UV72 1UV58 1UU84 1UU80 1UU72 1UU53 1UT84 1UT80 1UT72LA 1UT53 1US80 1US72 1US58 1UK93 1UK87 1UK80 1UK75 1UK74 1UK73LA 1UK72LA 1UK53 1UJ93 1UJ87 1UJ82 1UJ80 1UJ75 1UJ74 1UJ73LA 1UJ71 1UJ58 1UG93 1UG87 1UG80 1UG75 1UG74 1UG73LA 1UG72LA 1UG72JAHB 1UG72JAAZ 1UG57 1UG53 1UF93 1UF87 1UF84 1UF82 1UF80 1UF79 1UF74 1UF73LA 1UC89 1UC87 1UC82 1UC80 1UC79 1C75 1UC74 1UC73LA 1UC72 1UC57 1UC53 1UB93 1UB87 1UB83 1UB80 1UB75 1UB74 1UB73LA 1UB72LA 1UB72DA 1UB58 1UB57 1UB53 1TZ94LA 1TZ70LA 1TV93 1TV91 1TV87 1TV84 1TV83 1TV82 1TV80 1TV79 1TV74 1TV73LA 1TV58 1TS80 1TS72 1TS58 1TQ80 1TQ72 1TQ58 1TQ57 1TM93 1TM87 1TM83 1TM80 1TM75 1TM74 1TM73LA 1TM72LA 1TM72DA 1TM58 1TM57 1TM53 1TK93 1TK91 1TK87 1TK83 1TK82 1TK80 1TK79 1TK74 1TK73LA 1TK58 1TH 1TF80 1TF72 1TF58 1TF57 1TC80 1TC72 1TC57 1TB87 1TB80 1TB74 1TB72LA 1TB72JAHB 1TB72JAAZ 1TB72DA 1TA93 1TA87 1TA83 1TA80 1TA75 1TA74 1TA73LA 1TA72LA 1TA72DA 1TA58 1TA57 1TA53 1SY87 1SY84 1SY80 1SY72 1SY58 1SY57 1SY53 1SW87 1SW74 1SQ93 1SQ91 1SQ87 1SQ83 1SQ80 1SQ74 1SQ58 1SQ53 1SN93 1SN87 1SN75 1SN74 1SN72 1SN58 1SM87 1SM80 1SM74 1SM73LA 1SL91 1SL89 1SL87 1SL80 1SL74 1SL73LA 1SL58 1SK87 1SK80 1SK74 1SK73LA 1SG87 1SG80 1SG72WK 1SG72WJ 1SG58 1SF89 1SF87 1SF80 1SF75 1SF74 1SF73PF 1SE89 1SE87PF 1SE59LAGX 1SE53 1SC89 1SC87 1SC80 1SC75 1SC74 1SC72JAHB 1SC72JAAZ 1SA89 1SA80 1SA75 1SA74 |
| Neurosurgery | 1AA53SZPL 1AA80 1AA87 1AB86 1AB87 1AC50 1AC52 1AC53 1AC59 1AC87 1AE53 1AE59 1AE85 1AE87 1AF59 1AF87 1AG87 1AJ53 1AJ87 1AN53 1AN56 1AN59 1AN70 1AN73 1AN87 1AP52 1AP53 1AP59 1AP72 1AP87 1AW59 1AW72 1AW87 1AX52MESJ 1AX52MQSJ 1AX53 1AX56 1AX73 1AX80 1AX86 1AX87 1AZ94 1BA53SZDV 1BA59 1BA72 1BA80 1BA87 1BB53 1BB58 1BB59 1BB72 1BB80 1BB87 1BD58 1BD59 1BD72 1BD80 1BD87 1BF 1BG 1BJ53 1BJ59 1BK59 1BM 1BN 1BP 1BQ 1BS58 1BS59 1BS72 1BS80 1BS87 1BT 1BX53 1BX59 1BX72 1BX80 1BX87 1BZ 1JW50 1JW51 1JW57 1JW59 1JW76 1JW89 1JW86 1JW87 |
| Ophthalmologic | 1CC58 1CC59JAGX 1CC80LAXXA 1CC80LAXXK 1CC80LAXXQ 1CC84 1CC85 1CC87 1CD52 1CD53 1CD80LAXXA 1CD80LAXXK 1CD87 1CE56LA 1CE56LALZ 1CF59LAGX 1CF87 1CF91 1CG59 1CG71 1CG76 1CG87 1CH59LAGX 1CH72 1CH80 1CH87 1CJ56 1CJ59LAGX 1CJ80 1CJ87 1CL53 1CL56 1CL59 1CL87 1CL89 1CM59LA 1CM89 1CN59LAGX 1CN59LAGY 1CN72 1CP53 1CP56 1CP80 1CP87 1CP89 1CP91 1CQ59LAGX 1CQ72 1CQ78 1CQ80 1CQ83 1CQ87 1CR80 1CR87 1CS56LA 1CS56LALZ 1CS59JAGX 1CS72 1CS80 1CS84 1CS87 1CT51 1CT59 1CT80 1CT87 1CT89 1CU50 1CU51 1CU56 1CU57 1CU59LAGX 1CU72 1CU76LA 1CU76LANR 1CU76ML 1CU76MLNR 1CU80 1CU87 1CU89 1CV 1CX56LA 1CX59JAGX 1CX72 1CX74 1CX78 1CX80 1CX84 1CX87 1CX88 1CZ56LA 1CZ70 1CZ94HA 1CZ94LA |
| Peritoneal Dialysis Catheter | 1OT53 1OT54 1YY53LATS 1SY55 |
| Retroperitoneal | 1PB87 1PB89 1PC51LALV 1PC56 1PC59DAGX 1PC59LAGX 1PC80 1PC82 1PC83 1PC86 1PE50DABD 1PE50DABF 1PE50DABJ 1PE56DA 1PE56LA 1PE59LAAG 1PE59LAGX 1PE76 1PE77 1PE80 1PE82 1PE87 1PE89 1PG50DABD 1PG50DABF 1PG50DABJ 1PG50LABJ 1PG52DA 1PG56DA 1PG56LA 1PG57DAGX 1PG57LAAM 1PG57LAGX 1PG59DAAG 1PG59DAAS 1PG59DAAT 1PG59DAAZ 1PG59DAGX 1PG59KQAP 1PG59KQAQ 1PG59KQAR 1PG59LAAG 1PG59LAGX 1PG72 1PG74 1PG76 1PG77 1PG80DA 1PG80LA 1PG80LAXXE 1PG80LD 1PG82 1PG86 1PG87 1PG98 1PL50LAGX 1PL53 1PL59LAAD 1PL59LAAG 1PL59LAAS 1PL59LAAZ 1PL59LAGX 1PL72LA 1PL72LAAG 1PL74 1PL80 1PL87 1PM56DA 1PM56LA 1PM57DAGX 1PM57LAGX 1PM58LA 1PM59DAAG 1PM59DAAS 1PM59DAAT 1PM59DAAZ 1PM59DAGX 1PM59DAX7 1PM59KQAP 1PM59KQAQ 1PM59KQAR 1PM72 1PM77 1PM79 1PM80AF 1PM80FJ 1PM80LA 1PM82 1PM84 1PM86 1PM87LA 1PM89LA 1PM90 1PM91 1PM92 |
| Skin and Soft Tissue | 1MD87 1MD89 1MK87 1MK89 1MR50 1MR51 1MR52HA 1MR59 1MR76 1MR80 1MR87 1MR91 1MS50 1MS51 1MS52 1MS59 1MS76 1MS80 1MS87 1MS91 1SH56LA 1SH59 1SH87 1SZ56LA 1SZ59 1SZ87 1TX56LA 1TX59LA 1TX59LAGX 1TX87 1UY56LA 1UY57 1UY59 1UY72 1UY80 1UY87 1VX56LA 1VX59LAGX 1VX87 1WV56LA 1WV57 1WV58 1WV59LAGX 1WV72 1WV80 1WV87 1YA14JAXXK 1YA14JAXXL 1YA14JAXXN 1YA14JAXXP 1YA53 1YA58 1YA59JAGX 1YA59JALV 1YA80 1YA83 1YA87 1YB14JAXXK 1YB14JAXXL 1YB14JAXXN 1YB14JAXXP 1YB53 1YB58 1YB59JAGX 1YB74 1YB80LAXXA 1YB80LAXXB 1YB80LAXXE 1YB80LAXXF 1YB87 1YC14JAXXK 1YC14JAXXL 1YC14JAXXN 1YC14JAXXP 1YC58LAXXA 1YC59JAGX 1YC80LAXXA 1YC80LAXXB 1YC80LAXXE 1YC87 1YD14JAXXK 1YD14JAXXL 1YD14JAXXN 1YD14JAXXP 1YD59JAGX 1YD80LAXXA 1YD80LAXXB 1YD80LAXXE 1YD80LAXXF 1YD87 1YE14JAXXK 1YE14JAXXL 1YE14JAXXN 1YE14JAXXP 1YE59JAGX 1YE74 1YE78 1YE79LAXXA 1YE79LAXXL 1YE79LAXXN 1YE80LAXXA 1YE80LAXXB 1YE80LAXXE 1YE87 1YF14JAXXK 1YF14JAXXL 1YF14JAXXN 1YF14JAXXP 1YF53 1YF57JACF 1YF58 1YF59JAGX 1YF74 1YF80LAXXA 1YF80LAXXB 1YF80LAXXE 1YF80LAXXF 1YF87 1YG14JAXXK 1YG14JAXXL 1YG14JAXXN 1YG14JAXXP 1YG53 1YG58 1YG59JAGX 1YG74 1YG80LAXXA 1YG80LAXXB 1YG80LAXXE 1YG80LAXXF 1YG87 1YR14JAXXK 1YR14JAXXL 1YR14JAXXN 1YR14JAXXP 1YR59JAGX 1YR78LA 1YR78LAAZ 1YR80LAXXA 1YR80LAXXB 1YR80LAXXE 1YR80LAXXF 1YR87 1YS14JAXXK 1YS14JAXXL 1YS14JAXXN 1YS14JAXXP 1YS53 1YS58 1YS59JAGX 1YS74 1YS78LA 1YS78LAAZ 1YS80 1YS87 1YT14JAXXK 1YT14JAXXL 1YT14JAXXN 1YT14JAXXP 1YT53 1YT58 1YT59JAGX 1YT78HA 1YT78LA 1YT78LAAZ 1YT80 1YT87 1YU14JAXXK 1YU14JAXXL 1YU14JAXXN 1YU14JAXXP 1YU53 1YU58 1YU59JAGX 1YU80LAXXA 1YU80LAXXB 1YU80LAXXE 1YU80LAXXF 1YU80LAXXG 1YU87 1YV14JAXXK 1YV14JAXXL 1YV14JAXXN 1YV14JAXXP 1YV53 1YV58 1YV59JAGX 1YV74 1YV78LA 1YV78LAAZ 1YV80LAXXA 1YV80LAXXB 1YV80LAXXE 1YV80LAXXF 1YV87 1YW14JAXXK 1YW14JAXXL 1YW14JAXXN 1YW14JAXXP 1YW53 1YW58 1YW59JAGX 1YW80LAXXA 1YW80LAXXB 1YW80LAXXE 1YW80LAXXF 1YW87 1YX80 1YX87 1YX89 1YY14JAXXK 1YY14JAXXL 1YY14JAXXN 1YY14JAXXP 1YY80 1YY87 1YY87 1YY89 1YZ14JAXXK 1YZ14JAXXL 1YZ14JAXXN 1YZ14JAXXP 1YZ53 1YZ58 1YZ59JAGX 1YZ59JALV 1YZ78LA 1YZ78LAAZ 1YZ80LAXXA 1YZ80LAXXB 1YZ80LAXXE 1YZ80LAXXF 1YZ87 1YZ94 1YZ94LA |
| Thoracic | 1GM50LA 1GM56LA 1GM80DAXXE 1GM80LA 1GM80LAXXE 1GM80LAXXG 1GM86 1GM87 1GN 1GR 1GT56 1GT58 1GT59 1GT78 1GT80 1GT85 1GT87 1GT89 1GT91 1GV56 1GV59DAGX 1GV59DAZ9 1GV59LAGX 1GV76 1GV80 1GV87 1GV89 1GW56 1GW59 1GW87 1GX78 1GX80 1GX86 1GX87 1GY56 1GY70 1GY72 1GY86 1GY94DA 1GY94LA 1ME87 1ME89 1MF87 1MM 1MN50 1MN51 1MN59 1MN74 1MN76 1MN77 1MN80 1MN87 1NA56DB 1NA56DBXXF 1NA56DBXXG 1NA56EZ 1NA56EZXXG 1NA56FA 1NA56FAXXF 1NA56FAXXG 1NA56LB 1NA56LBXXF 1NA56LBXXG 1NA56LP 1NA56LPXXF 1NA56LPXXG 1NA56QB 1NA56QBXXF 1NA56QBXXG 1NA56QFXXF 1NA56QFXXG 1NA72 1NA74 1NA76 1NA77 1NA80 1NA82 1NA84 1NA86 1NA87 1NA88 1NA89 1NA90 1NA91 1NA92 |
| Vascular (excluding fistula procedures) | 1ID57 1ID76 1ID80 1ID82 1ID86 1ID87 1JD53 1JD59 1JD89 1JE50 1JE51 1JE57 1JE58 1JE59 1JE76 1JE80 1JE87 1JJ50 1JJ51 1JJ57 1JJ58 1JJ76 1JJ80 1JJ83 1JJ87 1JK50 1JK51 1JK57 1JK58 1JK76 1JK80 1JK87 1JL50 1JL51 1JL57 1JL58 1JL80 1JL87 1JQ50 1JQ51 1JQ57 1JQ80 1JQ87 1JT50 1JT51 1JT57 1JT58 1JT80 1JT87 1JU 1JY50 1JY51 1JY57 1JY76 1JY80 1JY87 1KA50 1KA53 1KA57 1KA58 1KA76 1KA80 1KA82 1KA87 1KE50 1KE51 1KE57 1KE58 1KE76 1KE80 1KE87 1KG50 1KG51 1KG57 1KG58 1KG76 1KG80 1KG82 1KG87 1KQ 1KR34 1KR50 1KR51 1KR53 1KR57 1KR58 1KR59 1KR76 1KR78 1KR80 1KR83 1KR87 1KT50 1KT51 1KT58 1KT76 1KT80 1KT82 1KT87 1KV53 1KV80 1KX80 1KZ 1KY*  *IKY with the exception of arteriovenous fistula creation (in separate category) |

*Of note, obstetrical surgical procedures such as caesarian section were excluded. Anorectal, Breast, Lower Urologic and Gynecologic, Ophthalmology, Retroperitoneal, and Thoracic, were combined into one category in the model building procedures (i.e., as “low-risk other” category).

**Supplementary Table 5.** Estimated Sample Size Calculations using ‘pmsampsize’ in Stata software v17.0 and as suggested by Riley et al. (2020)^6, 7^

|  | **Input** | | | **Output** | |
| --- | --- | --- | --- | --- | --- |
| **Sample Size Estimate Version** | **Estimated Outcome Prevalence and Source** | **R2 from literature^8^** | **Maximum Predictor Parameters** | **Sample Size** | **Events** |
| 1 | 1.7%^9^ | 0.072 | 32 | 3881 | 66 |
| 2 | 8.0%^8^ | 0.072 | 32 | 3838 | 308 |
| **Maximum** | **N/A** | **0.072** | **32** | **3881** | **308** |

**Supplementary Table 6.** Top causes of death for those that died within 30 days of surgery, with associated ICD-10 codes

| **Cause of Death** | **ICD-10 Death Code** | **No. (%)** |
| --- | --- | --- |
| Atherosclerotic Heart Disease | I251 | 64 (9) |
| Acute Myocardial Infarction Unspecified | I219 | 57 (8) |
| Vascular Disorder of Intestine not otherwise specified | K559 | 36 (5) |
| Chronic Renal Failure Unspecified | N189 | 33 (4) |
| Unspecified Diabetes without complications | E149 | 29 (4) |
| Unspecified diabetic nephropathy | E142 | 24 (3) |
| Unspecified Renal Failure | N19 | 22 (3) |
| Unspecified diabetic peripheral angiopathy | E145 | 18 (2) |
| Unspecified diabetic foot ulcer or other multiple complications | E147 | 16 (2) |
| Peripheral Vascular Disease Unspecified | I739 | 16 (2) |

**Supplementary Table 7.** Performance of models evaluated in cohort with only first surgery per participant

|  | **Model 1** | **Model 1 (first surgery)** | **Model 2** | **Model 2 (first surgery)** | **Model 3** | **Model 3 (first surgery)** |
| --- | --- | --- | --- | --- | --- | --- |
| *Number of surgeries included* | 38,541 | 8,977 | 38,541 | 8,977 | 38,541 | 8,977 |
| *Performance Metric* |  |  |  |  |  |  |
| C-statistic | 0.785 | 0.824 | 0.809 | 0.820 | 0.814 | 0.824 |
| Expected to Observed Ratio | 1.00 | 0.728 | 1.00 | 0.729 | 1.00 | 0.738 |
| Calibration intercept | 0.00 | 0.373 | 0.00 | 0.368 | 0.00 | 0.373 |
| Calibration slope | 1.00 | 0.95 | 1.00 | 0.945 | 1.00 | 0.95 |

**Supplementary Table 8.** Event and non-event Reclassification Tables between models, stratified by clinically important probability categories

**Model 2 versus Model 1**

|  |  | **Risk Model 2** Predicted Probability Threshold | | |  |  |  |  |
| --- | --- | --- | --- | --- | --- | --- | --- | --- |
| Outcome Status | **Risk Model 1** Predicted Probability Threshold | <5% | 5-15% | ≥ 15% | Total | Correct Percentage Reclassification | Incorrect Percentage Reclassification | Net Reclassification |
| Present | < 5% | 457 | 55 | 1 | 513 | 16.8% | 7.7% | 9.1% |
|  | 5-15% | 31 | 301 | 146 | 478 |  |  |  |
|  | ≥ 15% |  | 62 | 151 | 213 |  |  |  |
|  | Total | 488 | 418 | 298 | 1204 |  |  |  |
| Absent | < 5% | 31083 | 681 |  | 31764 | 4.0% | 3.5% | 0.5% |
|  | 5-15% | 1049 | 2895 | 639 | 4583 |  |  |  |
|  | ≥ 15% |  | 443 | 547 | 990 |  |  |  |
|  | Total | 32132 | 4019 | 1186 | 37337 |  |  |  |
| Category-based Net Absolute Reclassification Index (NARI) | NARI = 7.8 per 1000 patients | | | | |  |  |  |

**Model 2 versus Model 3**

|  |  | **Risk Model 3** Predicted Probability Threshold | | |  |  |  |  |
| --- | --- | --- | --- | --- | --- | --- | --- | --- |
| Outcome Status | **Risk Model 2** Predicted Probability Threshold | <5% | 5-15% | ≥ 15% | Total | Correct Percentage Reclassification | Incorrect Percentage Reclassification | Net Reclassification |
| Present | < 5% | 461 | 27 |  | 488 | 6.8% | 5.3% | 1.5% |
|  | 5-15% | 20 | 343 | 55 | 418 |  |  |  |
|  | ≥ 15% |  | 44 | 254 | 298 |  |  |  |
|  | Total | 481 | 414 | 309 | 1204 |  |  |  |
| Absent | < 5% | 31674 | 458 |  | 32132 | 1.9% | 1.9% | 0% |
|  | 5-15% | 506 | 3276 | 237 | 4019 |  |  |  |
|  | ≥ 15% |  | 217 | 969 | 1186 |  |  |  |
|  | Total | 32180 | 3951 | 1206 | 37337 |  |  |  |
| Category-based Net Absolute Reclassification Index (NARI) | NARI = 0.8 per 1000 patients | | | | |  |  |  |

NARI is the Net Absolute Reclassification Index, and is calculated as:

(Proportion of reclassification for patients with events x event rate) + (proportion reclassification for patients without events x non-event rate) x 1000

**Supplementary Figure 1.** Decision Curve Analysis to estimate the net benefit of use of perioperative risk prediction models in ambulatory or inpatient elective surgery (sensitivity analysis)

REFERENCES

1. Austin PC, Daly PA, Tu JV: A multicenter study of the coding accuracy of hospital discharge administrative data for patients admitted to cardiac care units in Ontario. *Am Heart J,* 144**:** 290-296, 2002

2. Smilowitz NR, Gupta N, Guo Y, Berger JS, Bangalore S: Perioperative acute myocardial infarction associated with non-cardiac surgery. *Eur Heart J,* 38**:** 2409-2417, 2017 10.1093/eurheartj/ehx313

3. Ye Y, Larrat EP, Caffrey AR: Algorithms used to identify ventricular arrhythmias and sudden cardiac death in retrospective studies: a systematic literature review. *Ther Adv Cardiovasc Dis,* 12**:** 39-51, 2018 10.1177/1753944717745493

4. Tonelli M, Wiebe N, Fortin M, Guthrie B, Hemmelgarn BR, James MT, Klarenbach SW, Lewanczuk R, Manns BJ, Ronksley P, Sargious P, Straus S, Quan H, Alberta Kidney Disease N: Methods for identifying 30 chronic conditions: application to administrative data. *BMC Med Inform Decis Mak,* 15**:** 31, 2015 10.1186/s12911-015-0155-5

5. Tonelli M, Wiebe N, Kovesdy CP, James MT, Klarenbach SW, Manns BJ, Hemmelgarn BR, for the Alberta Kidney Disease N: Joint associations of obesity and estimated GFR with clinical outcomes: a population-based cohort study. *BMC Nephrology,* 20**:** 204, 2019 10.1186/s12882-019-1351-9

6. Riley RD, Ensor J, Snell KIE, Harrell FE, Jr., Martin GP, Reitsma JB, Moons KGM, Collins G, van Smeden M: Calculating the sample size required for developing a clinical prediction model. *BMJ,* 368**:** m441, 2020 10.1136/bmj.m441

7. Ensor J: PMSAMPSIZE: Stata module to calculate the minimum sample size required for developing a multivariable prediction model. *Statistical Software Components.* Boston College Department of Economics, 2018

8. Harrison T, Ronksley PE, James MT, Ruzycki SM, McCaughey D, Zarnke KB, Wick J, Hemmelgarn BR: Mortality and cardiovascular events in adults with kidney failure after major non-cardiac surgery: A population-based cohort study. *Kidney International Reports,* 6**:** S227, 2021 10.1016/j.ekir.2021.03.550

9. Harrison TG, Hemmelgarn BR, James MT, Manns BJ, Tonelli M, Brindle ME, McCaughey D, Ruzycki SM, Zarnke KB, Wick J, Ronksley PE: Association of Kidney Function With Major Postoperative Events After Non-Cardiac Ambulatory Surgeries: A Population-Based Cohort Study. *Ann Surg*, 2021 10.1097/SLA.0000000000005040
